# 1458. Endoscopic Transmission of New Delhi Metallo-β-lactamase Producing *Klebsiella pneumoniae* Through a Gastroscope Without an Elevator Channel

**DOI:** 10.1093/ofid/ofad500.1295

**Published:** 2023-11-27

**Authors:** Ann F Yang, Adrienne Sherman, Elizabeth Nazarian, Wolfgang Haas, Jason Mehr, Thomas Kirn, Steven Brant, Michele Pedrani, Keith S Kaye, Susan Boruchoff, John P Mills

**Affiliations:** Rutgers Robert Wood Johnson Medical School, New Brunswick, New Jersey; New Jersey Department of Health, Trenton, New Jersey; Wadsworth, Albany, NY; New York State Department of Health, Wadsworth, New York; New Jersey Department of Health, Trenton, New Jersey; Rutgers Robert Wood Johnson Medical School, New Brunswick, New Jersey; Robert Wood Johnson University Hospital, New Brunswick, New Jersey; Robert Wood Johnson University Hospital, New Brunswick, New Jersey; Rutgers Robert Wood Johnson Medical School, New Brunswick, New Jersey; Robert Wood Johnson University Hospital, New Brunswick, New Jersey; Rutgers Robert Wood Johnson Medical School, New Brunswick, New Jersey

## Abstract

**Background:**

Endoscopic transmission of carbapenem-resistant Enterobacterales (CRE) has mostly involved elevator channel-containing duodenoscopes. We describe a New Delhi metallo-β-lactamase producing *Klebsiella pneumoniae* (NDMKP) outbreak with genetic and epidemiological evidence of transmission via a non-therapeutic gastroscope.

**Methods:**

Endoscope sampling and cultures were performed using FDA protocols. Carbapenemase gene testing was performed at the New Jersey Department of Health. CRE rectal colonization screening and whole-genome sequencing (WGS) were performed at the New York State Department of Health’s Wadsworth Center.

**Results:**

Four NDMKP cases were identified from clinical cultures between March–April 2022 in patients without travel history. Three of four cases underwent ≥ 1 gastrointestinal endoscopic procedure within two weeks prior to their infection, including four shared devices. Cultures from shared endoscopes were negative. Among 18 patients exposed to shared endoscopes who underwent NDMKP colonization screening, 2/18 (11%) screened positive. All isolates were highly related by WGS, with a maximum of 23 single nucleotide polymorphism (SNP) difference. Audits of endoscope reprocessing and a transition to duodenoscopes with disposable end caps occurred by June 2022.

In October 2022 a post-endoscopy NDMKP infection (case 7) was identified with two SNPs shared with case #2 (in March 2022). Two shared gastroscopes were identified with case #2 (GIF-1TH190 and GIF-H190); cultures from both were negative. GIF-H190 is a non-therapeutic gastroscope without an elevator channel. Four repairs to GIF-H190 for fluid intrusion or device buckling occurred from January–August 2022. Of 49 patients exposed to GIF-H190 who underwent NDMKP colonization screening; 1/49 (2%) was positive, with 14 SNPs from case #2. Boroscopic evaluation of endoscopes was initiated and repair work was transitioned from a third party to the device manufacturer. No further NDMKP cases have been identified to date.

**Figure 1:**
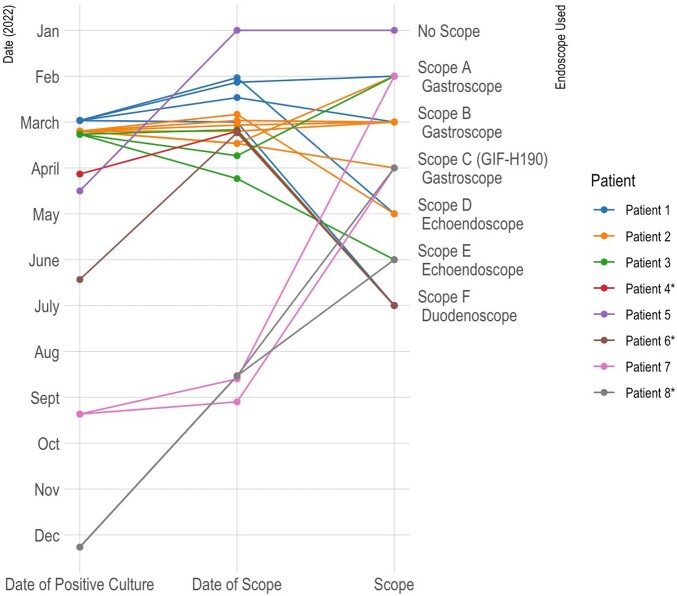
Timeline of Endoscopic Procedures and Positive NDMKP Cultures

Each line represents a scope, each color represents a patient. Left axis indicates date, right axis indicates which scope was used. *Patients identified via rectal swab screen, without clinical signs of infection.

**Figure 2:**
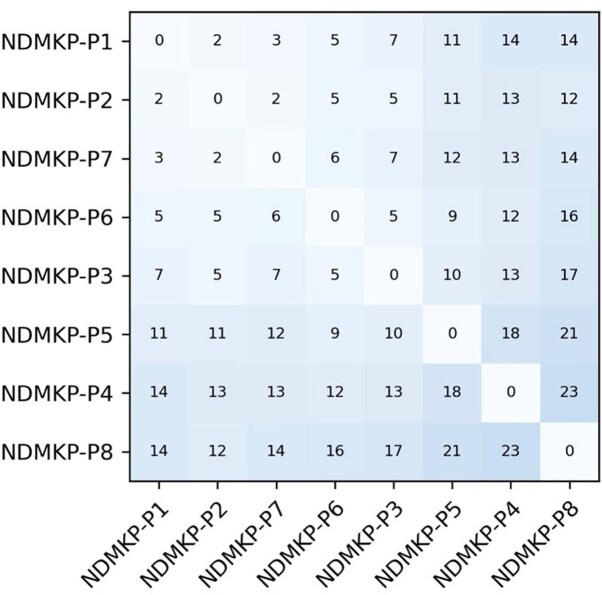
2 x 2 Table of Single Nucleotide Polymorphisms (SNP) by Whole Genome Sequencing for NDMKP isolates from Patients 1 - 8.

**Conclusion:**

Non-therapeutic gastroscopes without elevator channels may pose a risk for CRE transmission, particularly in the setting of mechanical damage. Reprocessing programs should focus on measures to promptly identify and repair physical damage to endoscopes.

**Disclosures:**

**Steven Brant, MD**, Bristol Myers Squibb: Research Grant on Education Hispanic population in inflammatory bowel disease **Keith S. Kaye, MD, MPH**, Abbvie: Advisor/Consultant|Abbvie: Honoraria|Entasis: Advisor/Consultant|Entasis: Honoraria|GSK: Advisor/Consultant|GSK: Honoraria|Merck: Advisor/Consultant|Merck: Honoraria|Shionogi: Advisor/Consultant|Shionogi: Honoraria|VenatoRx: Advisor/Consultant|VenatoRx: Honoraria

